# Clinical efficacy, safety, and predictors of treatment response to SGLT2 versus DPP-4 inhibitors in type 2 diabetes: a retrospective comparative study

**DOI:** 10.3389/fendo.2026.1818019

**Published:** 2026-05-01

**Authors:** Xinfeng Yao, Dongju Huang, Xiangying Shi, Fangping Xiang, Liu Liu

**Affiliations:** 1 Department of Endocrinology, Zhebei Branch of the Third Affiliated Hospital of Zhejiang Chinese Medical University, Wuxing District Hospital of Traditional Chinese Medicine, Huzhou, Zhejiang, China; 2 Department of Gastroenterology, Zhebei Branch of the Third Affiliated Hospital of Zhejiang Chinese Medical University, Wuxing District Hospital of Traditional Chinese Medicine, Huzhou, Zhejiang, China; 3 Department of Nephrology, Zhebei Branch of the Third Affiliated Hospital of Zhejiang Chinese Medical University, Wuxing District Hospital of Traditional Chinese Medicine, Huzhou, Zhejiang, China

**Keywords:** clinical efficacy, dipeptidyl peptidase 4 inhibitors, predictive factors of treatment response, safety, sodium-glucose cotransporter 2 inhibitors, type 2 diabetes

## Abstract

**Background:**

The prevalence of type 2 diabetes is continuously increasing, and optimizing the treatment strategies for diabetes is an important clinical issue. SGLT2 inhibitors (SGLT2i) and DPP-4 inhibitors (DPP-4i) are two commonly used oral hypoglycemic drugs, and their comprehensive efficacy and safety profiles in real-world settings merit in-depth comparison.

**Objective:**

To compare the clinical efficacy and safety of SGLT2i and DPP-4i in treating patients with type 2 diabetes from May 2024 to August 2025, and to explore the predictive factors influencing treatment response.

**Method:**

A retrospective cohort study was conducted using medical records of 100 patients with type 2 diabetes who met standardized screening criteria. These patients were assigned to two treatment groups: the SGLT2 inhibitor group (SGLT2i, n=50) and the DPP-4 inhibitor group (DPP-4i, n=50). Primary endpoints comprised changes in glycated hemoglobin (HbA1c) levels and the occurrence of major adverse cardiovascular events. Secondary outcomes including changes in body mass index, blood pressure, renal function parameters (urinary albumin-to-creatinine ratio and estimated glomerular filtration rate), lipid profile, liver function markers, and the frequency of adverse events. Exploratory measures, including inflammatory markers such as C-reactive protein, were also evaluated. Statistical analyses were performed using SPSS, including t-tests, chi-square tests, and multivariate logistic regression.

**Results:**

Regarding primary outcomes, comparable reductions in HbA1c were observed in both groups. In terms of safety profiles, the SGLT2i group exhibited a significantly lower rate of major adverse cardiovascular events compared to the DPP-4i group (2.00% vs. 16.00%, *P* < 0.05). A higher incidence of genital infections was noted in the SGLT2i group (14.00% vs. 2.00%), whereas no statistically significant difference was found in hypoglycemia events between the two groups. For secondary outcomes, patients receiving SGLT2 inhibitors demonstrated significantly greater improvements in body weight reduction, systolic blood pressure lowering, and urinary albumin-to-creatinine ratio compared to the DPP-4i group (*P* < 0.05). Multivariate Logistic regression analysis showed that SGLT2 inhibitor treatment (OR = 1.747, 95% CI: 1.137 - 2.684, *p* = 0.011), diastolic blood pressure (OR = 0.827, 95% CI: 0.694 - 0.985, *p* = 0.033), history of hypertension (OR = 0.010, 95% CI: 0.001 - 0.121, *p* < 0.001), and total bilirubin (OR = 1.956, 95% CI: 1.091 - 3.507, *p* = 0.024) were all independent influencing factors for the glucose treatment response.

**Conclusion:**

In patients with type 2 diabetes, SGLT2i and DPP-4i have comparable hypoglycemic efficacy, but SGLT2i shows additional advantages in weight loss, reduction in blood pressure, and improvement in renal outcomes. Baseline characteristics of patients may serve as useful references for individualized treatment choices.

## Highlights

This study innovatively combines multi-dimensional assessment results with treatment response prediction. By establishing a comprehensive assessment framework, it integrates key indicators (such as blood glucose control and cardiovascular events) with secondary indicators (such as weight, blood pressure, and renal function). It not only compares the efficacy and safety of drugs, but also identifies key predictive factors - particularly the baseline body mass index and the level of urinary albumin excretion rate - which have a significant impact on the treatment response of SGLT2i. This approach provides a new strategy for personalized drug selection in the management of type 2 diabetes, going beyond the traditional “one-size-fits-all” model. It provides a basis for evidence-based clinical decision-making, enabling treatment choices to be matched to individual patient characteristics.

## 1 Introduction

Type 2 diabetes, as one of the most common chronic metabolic diseases worldwide, has seen a continuously rising prevalence, which has become a serious public health challenge (1). According to epidemiological surveys, approximately 500 million adults worldwide suffer from diabetes, among whom type 2 diabetes accounts for over 90% of the total cases (2). The condition is characterized by chronic hyperglycemia and is associated with a spectrum of complications, including microvascular disorders such as nephropathy, retinopathy, and neuropathy, as well as macrovascular events affecting the cardiovascular and cerebrovascular systems. These serious complications not only significantly impair patients’ quality of life and reduce life expectancy but also place a substantial strain on healthcare resources (3, 4).

In the long-term management strategy for type 2 diabetes, maintaining blood sugar control has always been the core therapeutic goal. However, modern treatment concepts have shifted from merely managing blood sugar to a patient-centered, comprehensive management model that emphasizes controlling blood sugar while also addressing multiple risk factors such as weight, blood pressure, and blood lipids, and striving to reduce the risk of cardiovascular events and microvascular complications (5). The drug treatment plans have also been continuously evolving, moving from traditional medications such as metformin and sulfonylureas to the various new hypoglycemic drugs that have emerged in recent years, providing the clinical setting with a much broader range of treatment options (6, 7). Among these, sodium-glucose cotransporter 2 inhibitors (SGLT2i) and dipeptidyl peptidase-4 inhibitors (DPP-4i) represent two distinct classes of oral antihyperglycemic agents with established roles in clinical management (8).

DPP-4 inhibitors act by preventing the degradation of endogenous glucagon-like peptide-1, which in turn stimulates insulin secretion and inhibits glucagon release. Its glucose concentration-dependent hypoglycemic properties and low risk of hypoglycemia have made it favored in clinical applications (9, 10). The mechanism of SGLT2 inhibitors involves the inhibition of glucose reabsorption in the proximal renal tubules, promoting urinary glucose elimination and consequently lowering plasma glucose. Owing to this distinct, non-insulin-dependent action, these agents can achieve consistent glycemic reduction in patients with T2DM, irrespective of disease progression (11). Emerging evidence from a series of major cardiovascular outcome trials indicates that SGLT2 inhibitors confer cardiovascular benefits independent of glycemic control. Their pronounced advantages in reducing heart failure hospitalization risks and retarding renal dysfunction progression have markedly transformed the clinical management paradigm for type 2 diabetes (12, 13).

Although numerous randomized controlled trials have confirmed the efficacy and safety of these two types of drugs respectively, studies that directly compare their overall benefits in real-world clinical settings are still relatively limited (14). Randomized controlled trials, due to their rigorous design and control, have high internal validity. However, the populations they typically include are those that have been screened and are relatively homogeneous, which may not fully represent the diverse patient groups encountered in daily clinical practice. Most existing studies focus on limited indicators, such as blood glucose control and weight changes, and do not provide a systematic assessment of the comprehensive benefits of the two drug types across aspects such as blood pressure management, lipid improvement, and kidney protection (14). Meanwhile, when clinical doctors are formulating treatment plans for individual patients, they often need to answer the question “Which treatment is more suitable for this specific patient?” This requires a deeper understanding of the baseline factors that may predict the treatment response (15, 16).

Based on the aforementioned research background and the existing knowledge gaps, this study aims to systematically compare the clinical efficacy and safety of patients with type 2 diabetes who received SGLT2i or DPP-4i treatment from May 2024 to August 2025 through a retrospective cohort analysis. The primary study objectives encompassed several aspects. First, a comprehensive evaluation was conducted to compare key clinical outcomes—including changes in HbA1c and rates of major adverse cardiovascular events—between the two patient groups; second, to compare a series of secondary outcome indicators, covering multiple dimensions such as weight, blood pressure, lipid profile, and renal function indicators; third, to explore the baseline clinical characteristics that may predict treatment response, in order to identify which patients are more likely to benefit the most from a specific treatment.

The innovation of this study lies in its adoption of an integrated research perspective and methodological strategy. Firstly, we constructed a multi-dimensional evaluation framework that combines the main efficacy indicators with multiple secondary outcome indicators to conduct a more comprehensive evaluation of the “combined benefits” of the two types of drugs. Secondly, the study goes beyond the simple comparison of “which drug is better”, and is committed to exploring the sources of treatment response heterogeneity by analyzing the association between baseline characteristics and treatment outcomes, attempting to answer the more clinically practical question of “who is more likely to benefit more from which treatment”. Finally, this study focuses on recent real-world clinical data, which can reflect the actual effects of these two drug types in current clinical practice. The research findings of this study are expected to provide direct references for clinicians to formulate individualized treatment plans and promote the practical application of the precision medicine concept in the management of type 2 diabetes.

## 2 Research subjects and methods

### 2.1 Research subjects

**Figure 1:**
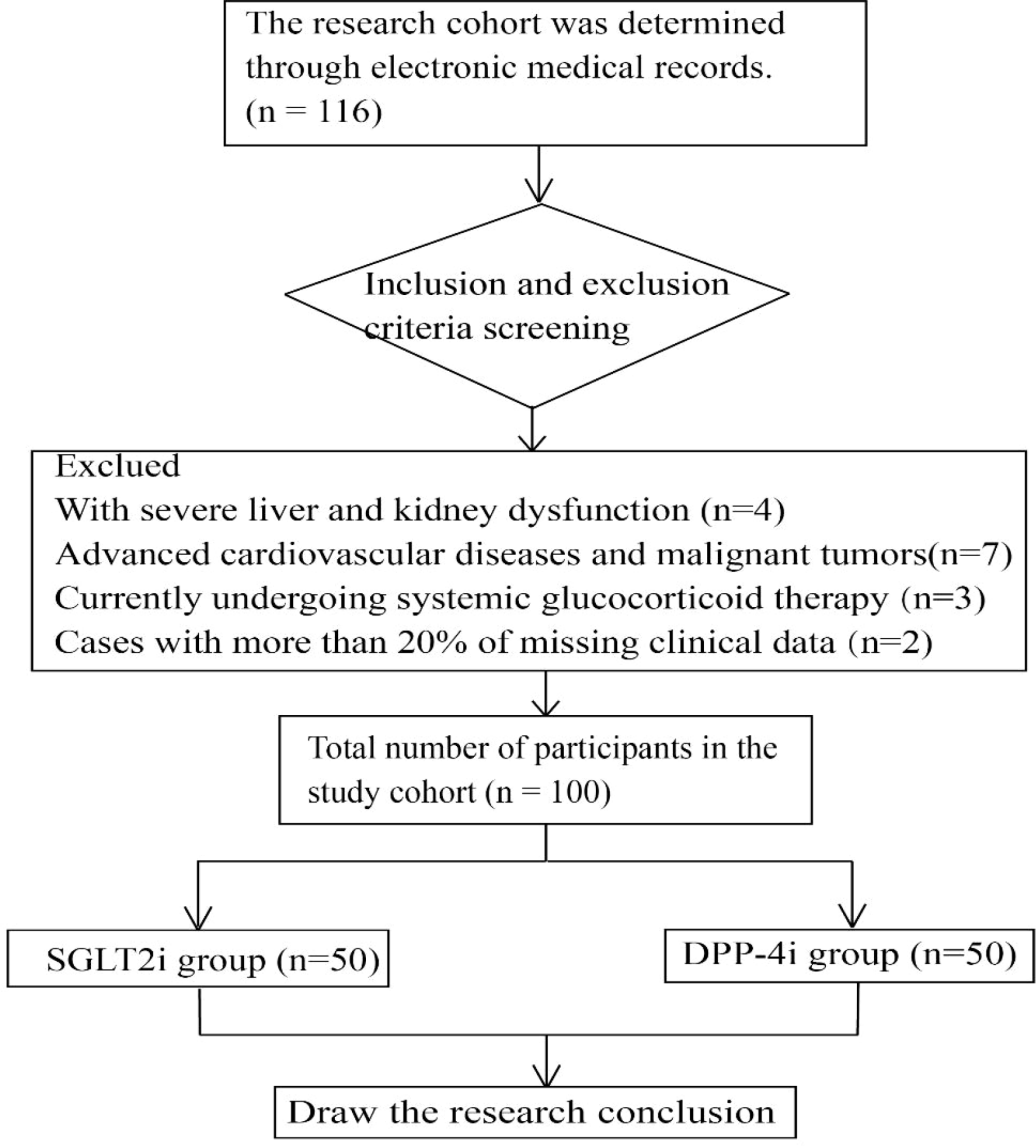
Flowchart. This study first identified a cohort of patients with type 2 diabetes who visited the endocrinology department of our hospital from May 2024 to August 2025 as the initial research population. Subsequently, through strict inclusion and exclusion criteria, 100 patients who met the requirements and had complete data were selected for analysis. These patients were further divided into SGLT2i group and the DPP-4i group based on their initial treatment regimens. By systematically collecting and statistically analyzing the baseline data, laboratory test results during treatment, and adverse event records of the two groups of patients, the clinical efficacy and safety were compared, and the predictive factors for treatment response were further explored.

This study enrolled 100 patients from the endocrinology department of our hospital between May 2024 and August 2025. Based on the initial therapeutic regimen, they were allocated to an SGLT2i group (n=50) or a DPP-4i group (n=50). Key baseline characteristics, such as age, gender, diabetes duration, and HbA1c levels, were similar between the groups, ensuring comparable baseline profiles. All these patients had varying degrees of poor glycemic control, and most of them presented with typical metabolic syndrome manifestations, including overweight, high blood pressure, abnormal lipid metabolism, and other common features. Some patients had early diabetic nephropathy or mild cardiovascular risk factors, which could well represent the characteristics of the common type 2 diabetes patients in clinical practice. All patients received at least 6 months of continuous medication treatment and regular follow-up. The complete clinical data of these patients provided a reliable database for this retrospective study. The diagnosis of type 2 diabetes is based on the standards released by the American Diabetes Association (ADA) in 2024. That is, any of the following conditions must be met and confirmed by a retest: fasting blood glucose ≥ 7.0 mmol/L, 2-hour blood glucose after oral glucose tolerance test ≥ 11.1 mmol/L, glycosylated hemoglobin ≥ 6.5%, or presence of typical hyperglycemic symptoms accompanied by random blood glucose ≥ 11.1 mmol/L (5).The patients included in this study all received SGLT2 inhibitors or DPP-4 inhibitors as the main hypoglycemic drugs. Among them, some patients received monotherapy, that is, at the time of enrollment or after conversion, they only used one of the above two types of drugs for hypoglycemic treatment; some patients received combination therapy, that is, on the basis of using SGLT2 inhibitors or DPP-4 inhibitors, they combined other hypoglycemic drugs, including metformin, sulfonylurea drugs, insulin, thiazolidinediones, α-glucosidase inhibitors, etc. The proportion of combined medication and the specific drug distribution in the two groups are shown in the baseline characteristics table. To ensure comparability between the groups, in the multivariate regression analysis, the combined medication situation (whether to combine other hypoglycemic drugs) was used as a covariate for correction. As shown in **Figure 1**.

### 2.2 Exclusion and inclusion criteria

Inclusion criteria (17): (1) Aged 30 to 75 years;(2) Confirmed diagnosis of type 2 diabetes;(3) Either naïve to SGLT2i or DPP-4i therapy, or switched from a prior glucose-lowering regimen to one of these agents;(4) Availability of complete follow-up data for a minimum of 6 months.

Exclusion criteria (18, 19): (1) Patients with type 1 diabetes, gestational diabetes, and other special types of diabetes; (2) Excluding those with severe liver or kidney dysfunction (specifically defined as transaminase levels exceeding three times the normal upper limit or estimated glomerular filtration rate lower than 30 mL/min/1.73m² (20, 21)); (3) Advanced cardiovascular diseases, malignant tumors; (4) Undergoing systemic glucocorticoid therapy; (5) Cases with missing clinical data exceeding 20%.

### 2.3 Treatment plan

This study was designed as a single-center, retrospective cohort analysis. Patient information, including baseline characteristics, laboratory parameters, and documented adverse events, was retrieved from the hospital’s electronic medical records. To ensure data integrity, a double-data entry process with cross-verification was implemented. The primary endpoint was the change from baseline in HbA1c at 6 months. Secondary endpoints included alterations in body weight, blood pressure, lipid profiles, renal function parameters, and the occurrence of adverse events.

As this study was of a retrospective design, the selection of drugs was based on clinical routine practice. The attending physicians made the decisions by comprehensively considering the baseline characteristics of the patients (including age, body mass index, blood pressure, renal function, cardiovascular risk, comorbidities, and previous medication history, etc.) and then formulated the treatment plans. To control for selection bias, we adjusted for the baseline differences between the groups through multivariate Logistic regression analysis to assess the differences in efficacy and safety of the two drugs as objectively as possible.

The concomitant medication usage of patients during treatment was retrospectively collected through the electronic medical record system, with a focus on two types of drugs that may affect cardiovascular outcomes: ① Statins (including atorvastatin, rosuvastatin, simvastatin, etc.); ② Renin-angiotensin system blockers (RAAS blockers), including angiotensin-converting enzyme inhibitors (ACEI) and angiotensin II receptor antagonists (ARB). The use of these drugs at baseline and during follow-up, as well as the types and duration of the medication, were recorded. In the multivariate analysis, the use of statins (yes/no) and the use of RAAS blockers (yes/no) were included as covariates in the Logistic regression model to control for their potential confounding effects on cardiovascular outcomes and treatment responses.

### 2.4 Efficacy evaluation criteria

In this study, “glycemic treatment response” was defined as a decrease in glycated hemoglobin (HbA1c) of ≥1.0% from baseline. This threshold was determined based on the following considerations: Firstly, from a clinical perspective, the UK Prospective Diabetes Study (UKPDS) and subsequent numerous large-scale clinical trials have demonstrated that for every 1% reduction in HbA1c, the risk of microvascular complications (such as retinopathy, nephropathy, and neuropathy) associated with diabetes can be reduced by approximately 30%-40%, the risk of myocardial infarction can be reduced by about 14%, and the risk of diabetes-related mortality can be reduced by about 21% (22). Therefore, a 1.0% reduction represents a clearly beneficial improvement. Secondly, from the perspective of study feasibility, the average baseline HbA1c level of patients in this study was around 8.5%, and a 1.0% reduction would result in a level of 7.5%. This target is both clinically challenging and within the reasonable treatment expectation range, effectively differentiating patients with good and poor treatment responses. Thirdly, from a methodological perspective, using an absolute reduction of ≥1.0% as the response criterion is more fair in evaluating the actual efficacy of the drug in patients with different baseline levels compared to using an absolute target of HbA1c < 7.0%. It avoids bias in the evaluation of efficacy due to baseline values. Moreover, this threshold has been widely adopted in numerous diabetes drug clinical trials and real-world studies both domestically and internationally, facilitating the comparison and dialogue of the study results with previous literature.

### 2.5 Detection indicators

#### 2.4.1 Primary efficacy indicators

1. The assay principle exploits the stable, non-enzymatic binding of glucose to the N-terminal valine of the hemoglobin beta chain. HbA1c levels were quantified using high-performance liquid chromatography (HPLC) based on this principle. Different charged hemoglobin components are separated by a cation exchange chromatography column, and the proportion of HbA1c in total hemoglobin is calculated. The measurement protocol was as follows: 2 mL of venous blood was drawn into an EDTA-K_2_ tube, gently inverted for mixing, and analyzed on an automated biochemistry analyzer. Internal quality control was maintained using two levels of commercial control materials. This method demonstrates high precision and accuracy, is considered the gold standard for assessing glycemic control, and reflects the patient’s average blood glucose levels over the preceding 8–12 weeks.

2. The major adverse cardiovascular events are evaluated according to standardized clinical diagnostic criteria. The diagnosis of myocardial infarction is based on the fourth edition of the global unified definition of myocardial infarction, which requires the presence of typical chest pain symptoms, characteristic dynamic changes in the electrocardiogram, and typical patterns of increase and decrease in myocardial enzyme levels (especially troponin) (23). The diagnosis of stroke is based on the standards set by the World Health Organization. It requires the presence of rapidly developing focal or generalized brain function impairment symptoms that persist for more than 24 hours, and which are confirmed by head CT or MRI (24). All events are independently reviewed and confirmed by the members of the Cardiovascular Event Determination Committee of this hospital, ensuring the accuracy and consistency of the diagnoses.

#### 2.5.2 Secondary efficacy indicators

1. Blood glucose measurement: The glucose oxidase method is used. After collecting venous blood, plasma is separated immediately, and the test is completed within 2 hours. A fasting blood glucose test requires patients to fast for at least 8 hours, and blood samples are collected early the next morning. Post-meal blood glucose is collected 2 hours after the first bite of food.

2. Lipid measurement uses enzymatic methods. Total cholesterol was quantified using commercial kits utilizing the cholesterol esterase-cholesterol oxidase-peroxidase system. Serum triglyceride levels were measured with the phosphoglycerol oxidase-based method. For high-density lipoprotein cholesterol, a homogeneous, selective inhibition assay was employed, whereas low-density lipoprotein cholesterol was assessed using a direct surfactant-clearance method. All tests are conducted after the patients have fasted for 12 hours, and venous blood is collected, and serum is separated within 4 hours for testing.

3. The measurement of body weight indicators is conducted using standardized anthropometric methods. The weight is measured using a calibrated electronic scale. The patient should wear light clothing and remove shoes, with the measurement accuracy to 0.1 kg. The height measurement is performed using a wall-mounted height gauge. The patient is required to have their heels together, stand upright, and keep their eyes level. We calculated body mass index using the standard formula: weight (kg) divided by height squared (m²). For waist circumference, a soft measuring tape was placed midway between the inferior margin of the last rib and the iliac crest. The patient was instructed to maintain a natural standing position and breathe quietly during the procedure (25).

4. Blood pressure was assessed in accordance with standardized clinic measurement guidelines. A validated electronic sphygmomanometer was used for all measurements. Participants were seated with their back supported and rested quietly for a minimum of five minutes prior to measurement. The cuff was applied directly to the bare upper arm, positioning its lower border 2–3 cm above the antecubital fossa. Three consecutive measurements are taken and the average is calculated. During the first visit, blood pressure on both sides is measured and the higher value is taken as the baseline. For subsequent follow-ups, blood pressure is measured on the same side of the arm (26).

5. Renal function indicators are measured through standardized laboratory tests. The urine albumin/creatinine ratio is measured by collecting the midstream urine in the morning. Urine albumin is detected using the immunoturbidimetric method, while urine creatinine is measured using the picric acid method. The ratio of the two is calculated. The estimated glomerular filtration rate was determined from serum creatinine using the CKD-EPI equation, which accounts for age, sex, and race (27).

6. Methods for detecting liver function indicators.

In this study, the detection of liver function indicators strictly followed the standardized laboratory operation procedures, and was measured using an automatic biochemical analyzer. Quantification of alanine aminotransferase, aspartate aminotransferase, and γ-glutamyl transferase was performed in accordance with the International Federation of Clinical Chemistry’s recommended reference procedure. Alkaline phosphatase was determined using the rate method with AMP buffer. Total bilirubin was determined using the diazotization method. Albumin was determined using the bromocresol green method. All tests were subject to internal quality control using two levels of quality control materials and participated in the inter-laboratory quality assessment organized by the National Health Commission Clinical Laboratory Center. All samples were tested within 2 hours after collection to avoid the influence of repeated freezing and thawing on enzyme activity.

7. The high-sensitivity C-reactive protein is based on the principle of immunosonic turbidimetry. It is based on the specific reaction between antigens and antibodies. In the presence of polyethylene glycol, C-reactive protein forms an immune complex with the specific antibody, resulting in turbidity. The intensity of this turbidity is proportional to the concentration of C-reactive protein. This method has a sensitivity of up to 0.1 mg/L and can detect low-level inflammatory conditions (28).

#### 2.5.3 Safety indicators

The analysis of hypoglycemic events follows the internationally accepted grading standards. Hypoglycemic events were classified into three grades: Grade 1 was characterized by a plasma glucose level ranging from 3.0 mmol/L to 3.9 mmol/L. A reading below 3.0 mmol/L indicated Grade 2. Grade 3 was designated for any severe event documented in the medical records where the patient required external assistance (29). All cases of hypoglycemia were determined based on multiple sources of information, such as patients’ self-monitoring records, data from the continuous glucose monitoring system, and emergency visit records.

2. The confirmation of genital infections is based on the clinical symptoms and laboratory test results recorded in the medical records. The diagnosis is based on the patient’s complaints recorded in the medical records (such as itching, pain, abnormal discharge, etc.) and the clinicians’ physical examination findings. In some cases, laboratory reports of fungal smears or bacterial cultures are also used as support. The diagnostic criteria for urinary tract infections include the presence of urinary tract irritation symptoms, positive routine urine test results for leukocyte esterase or nitrite, and, in some cases, laboratory evidence of a colony count greater than 10^^^5 CFU/ml from midstream urine culture (30).

3. The collection of safety data is achieved by systematically reviewing existing multi-source information such as medical records, laboratory test reports, emergency registration records, and follow-up files. Each adverse event is meticulously recorded for the time point of occurrence, severity classification, clinical treatment measures taken, and final outcome, providing comprehensive retrospective data to assess drug safety characteristics.

4. The primary adverse cardiovascular event (MACE) is defined as a composite endpoint, consisting of the following three core events: ① cardiovascular death; ② non-fatal myocardial infarction; ③ non-fatal stroke. All MACE events are determined based on medical records, and the diagnostic criteria are as follows: cardiovascular death refers to death directly caused by myocardial infarction, heart failure, stroke, or other cardiovascular causes; the diagnosis of non-fatal myocardial infarction is based on the fourth edition of the global unified definition of myocardial infarction, requiring an increase or decrease in myocardial necrosis markers (troponin) and at least one evidence of myocardial ischemia (such as typical chest pain symptoms, new ischemic changes on electrocardiogram, pathological Q wave formation, or new loss of myocardial viability shown by imaging); the diagnosis of non-fatal stroke is based on the World Health Organization standard, defined as acute focal or global brain functional impairment symptoms that last for more than 24 hours and confirmed as ischemic or hemorrhagic stroke by head CT or MRI, excluding transient ischemic attack. All MACE events are independently reviewed and confirmed by at least two members of the hospital’s cardiovascular event determination committee, and when opinions are inconsistent, consensus is reached through consultation.

### 2.6 Sample size calculation

This study was designed as a retrospective study. The study cohort size was defined as the number of individuals who were consecutively enrolled who met the inclusion and exclusion criteria over the course of the investigation. By systematically reviewing our hospital’s electronic medical record system, we screened patients with type 2 diabetes who visited the endocrinology department from May 2024 to August 2025. Eventually, 100 patients with complete baseline and follow-up data were selected and included in the final analysis. We performed a *post hoc* power calculation to assess the ability of the enrolled cohort to identify clinically relevant effects on the primary outcome measures. The sample size calculation was based on the previous research data of Xiaoyun Su et al. (31), with the 2-hour post-meal blood glucose of the DPP-4i group being 9.834 ± 1.75 and that of the SGLT2i group being 8.78 ± 1.27. The calculated effect size d was 0.69. The calculation was performed using the G*power software. Assuming a two-sided α=0.05 and β=0.20, the total sample size of 100 patients (50 per cohort) yielded a power of over 90%. This indicates that the study was well-powered to detect clinically significant differences in the primary efficacy measures, ensuring reliable statistical support for its findings.

### 2.7 Statistical methods

All statistical analyses were performed with SPSS version 25.0. Continuous variables, including age, disease duration, anthropometric measures, laboratory values (e.g., blood pressure, lipid profiles, renal and liver function tests, and inflammatory markers), are summarized as mean ± standard deviation and were compared between groups using the independent samples t-test. Categorical data, such as sex, comorbidities, and adverse event rates, are presented as counts (percentages) and were analyzed employing the χ² test or Fisher’s exact test, as appropriate. A multivariate logistic regression model was constructed with “glycemic treatment response,” defined as a reduction in HbA1c ≥ 1.0% from baseline, serving as the outcome variable. All tests were two-sided, and a *P* value of less than 0.05 was deemed statistically significant. To control the influence of potential confounding factors on the research results, the following covariates were included in the multiple regression analysis of this study: demographic characteristics (age, gender); clinical characteristics (duration of diabetes, baseline HbA1c, body mass index, systolic blood pressure, diastolic blood pressure); comorbidities (history of hypertension, history of dyslipidemia, history of diabetic nephropathy); concomitant medications (statins, RAAS blockers, the use of other hypoglycemic drugs in combination). Through multivariate adjustment, the interference of confounding factors on the main outcome indicators was as much as possible excluded.

### 2.8 Ethical statement

This research plan has been reviewed and approved by the ethics committee of our hospital.(Approval Date: October 15, 2025, Approval Number: ZSLL-KY-2025-108-01). As the research is a retrospective analysis, no intervention measures are involved, and all data are extracted after de-identification. The ethics committee has approved the exemption of patient informed consent. During the research process, the ethical principles of the Helsinki Declaration were strictly followed, and strict confidentiality measures were taken for all patients’ personal information and clinical data. The data extraction and analysis process was carried out in a controlled network environment within the hospital, and all electronic data were anonymized and encoded to ensure that no personal identity could be traced. The research results are presented only in summary form, and no information that could identify individuals will be disclosed.

## 3 Results

### 3.1 Population baseline characteristics

The cohort comprised 100 patients with type 2 diabetes, distributed equally between the SGLT2i and DPP-4i groups. Comparative analysis of baseline demographics and clinical features (**Table 1**) indicated that the two groups were well matched, with no significant differences in age, gender, disease duration, anthropometrics, blood pressure, or comorbidity profiles (all *P* > 0.05). Specifically, the average age of patients in both groups was approximately 58 years, and the proportion of males was comparable (56.0% vs 50.0%). The average duration of diabetes was approximately 8.5 years, indicating a medium to long-term disease course. The body mass index was above 28 kg/m², meeting the criteria for overweight; blood pressure levels were within the range of normal high values or grade 1 hypertension. The analysis of comorbidities showed that approximately 62% vs 60% of patients had hypertension, approximately 70% vs 66% had dyslipidemia, and approximately 30% vs 26% had developed diabetic nephropathy. The balance of all baseline characteristics indicated that the two groups of patients were well comparable, providing a reliable basis for comparing subsequent treatment effects.

**Table 1:** Compare the demographic characteristics of the participants.

Baseline		SGLT2i group (n = 50)	DPP-4i group (n = 50)	Test	95%CI		Effect size	*t*	*P*-value
					Lower	Upper			
Age		58.30 ± 8.74	57.62 ± 9.17	T test	-2.876	4.236	0.171	0.379	0.705
Gender (n, %)	Male	28 (56.00)	25 (50.00)	Chi-squared			0.361		0.548
	Female	22 (44.00)	25 (50.00)						
Duration of diabetes (years)		8.53 ± 3.21	8.82 ± 3.51	T test	-1.617	1.057	1.149	-0.416	0.678
Weight (kg)		78.54 ± 6.82	77.81 ± 6.50	T test	-1.906	3.385	0.429	0.555	0.580
BMI (kg/m², $\bar{x}$ ± s)		28.66 ± 2.25	28.11 ± 2.21	T test	-0.334	1.436	0.199	1.235	0.220
Systolic blood pressure (mmHg, $\bar{x}$ ± s)		137.66 ± 8.53	138.60 ± 8.31	T test	-4.281	2.402	0.069	-0.558	0.578
Diastolic blood pressure (mmHg, $\bar{x}$ ± s)		86.09 ± 5.42	86.14 ± 5.03	T test	-2.122	2.030	0.637	-0.044	0.965
Total Cholesterol ( $\bar{x}$ ± s)	xT_0_	5.23 ± 0.58	5.08 ± 0.62	T test	-0.098	0.379	0.638	1.170	0.245
History of hypertension (n, %)	No	19 (38.00)	20 (40.00)	Chi-squared			0.042		0.838
	Yes	31 (62.00)	30 (60.00)						
History of dyslipidemia (n, %)	No	15 (30.00)	17 (34.00)	Chi-squared			0.184		0.668
	Yes	35 (70.00)	33 (66.00)						
Diabetic nephropathy (n, %)	No	35 (70.00)	37 (74.00)	Chi-squared Chi-squared			0.198		0.656
	Yes	15 (30.00)	13 (26.00)						

BMI, Body Mass Index.

### 3.2 Main outcome

The two groups of patients showed similar improvement trends in glucose metabolism indicators (**Table 2**). Following the 6-month treatment period, significant reductions from baseline were observed in HbA1c, fasting blood glucose, and 2-hour postprandial glucose levels within each group (all *P* < 0.05). However, the extent of improvement in these glycemic parameters did not differ significantly between the SGLT2i and DPP-4i groups (*P* > 0.05), suggesting similar glucose-lowering efficacy between the two regimens.

**Table 2:** Changes in blood sugar levels ( $\bar{x}$ ± s).

Parameters		SGLT2i group (n = 50)	DPP-4i group (n = 50)	Test	95%CI		Effect size	t	*P*-value
Glycated hemoglobin (%)	T_0_	8.52 ± 0.81	8.41 ± 0.72	T test	-0.204	0.404	0.036	0.654	0.515
	T_1_	6.92 ± 1.62^*^	5.92 ± 1.32^*^	T test	-0.107	0.307	2.201	0.961	0.339
Fasting blood glucose (mmol/L)	T_0_	9.84 ± 1.18	9.64 ± 1.105	T test	-0.255	0.654	0.607	0.872	0.385
	T_1_	7.48 ± 0.93	7.43 ± 0.75	T test	-0.297	0.377	0.842	0.237	0.813
PBG (mmol/L)	T_0_	13.51 ± 2.08	13.32 ± 2.01	T test	-0.631	0.992	0.492	0.441	0.660
	T_1_	9.78 ± 1.51	9.63 ± 1.37		-0.414	0.734	1.980	0.552	0.582

HbA1c, Glycated hemoglobin; PBG, Two-hour post-meal blood glucose level. T_0_ is Baseline period. T_1_ is Six months after the treatment.

The overall adverse event profile was similar between the two treatment groups (**Table 3**). A notably higher incidence of genital infections was observed in the SGLT2i cohort compared with the DPP-4i group (14.0% vs. 2.0%, P < 0.05), consistent with the established side-effect profile of this drug class. Conversely, the SGLT2i group demonstrated a significantly lower rate of adverse cardiovascular events (*P* < 0.05). The incidence of hypoglycemia did not differ significantly between groups, confirming the glucose-dependent safety mechanism common to both medications.

**Table 3:** Incidence rate comparison of adverse events (n, %).

Type of adverse event	SGLT2i group (n = 50)	DPP-4i group (n = 50)	Test	Effect size	df	*P*-value
Hypoglycemic event	3 (6.00)	4 (8.00)	Chi-squared	0.154	1	0.695
Genital infection	7 (14.00)	1 (2.00)	Chi-squared	4.895	1	0.027
Urinary tract infection	2 (4.00)	1 (2.00)	Chi-squared	0.344	1	0.558
Adverse cardiovascular events	2 (2.00)	8 (16.00)	Chi-squared	4.000	1	0.046
Other adverse events	3 (6.00)	2 (4.00)	Chi-squared	0.211	1	0.645
Total incidence rate of adverse events	16 (32.00)	16 (32.00)	Chi-squared	0.000	1	1.000

### 3.3 Secondary outcome indicators

In terms of lipid improvement (**Table 4**), there were no significant differences between the two groups in changes in total cholesterol, LDL-C, and HDL-C (*P* > 0.05). Notably, the SGLT2i group showed a significantly greater reduction in triglycerides compared to the DPP-4i group (P < 0.05), indicating a greater improvement in triglycerides.

**Table 4:** Changes in lipid profile indicators of the two groups (mmol/L, $\bar{x}$± s).

Parameters		SGLT2i group (n = 50)	DPP-4i group (n = 50)	Test	95%CI		Effect size	t	*P*-value
Total Cholesterol ( $\bar{x}$ ± s)	T_0_	5.23 ± 0.58	5.08 ± 0.62	T test	-0.098	0.379	0.638	1.170	0.245
	T_1_	4.93 ± 0.54	4.84 ± 0.53	T test	-0.120	0.300	0.034	0.851	0.397
Triglyceride	T_0_	2.34 ± 0.51	2.22 ± 0.49	T test	-0.088	0.309	0.515	1.104	0.272
	T_1_	1.96 ± 0.44	2.15 ± 0.37	T test	-0.353	-0.028	3.529	-2.310	0.023
LDL-C	T_0_	3.16 ± 0.48	3.14 ± 0.53	T test	-0.171	0.230	1.007	0.293	0.770
	T_1_	2.91 ± 0.44	2.91 ± 0.38	T test	-0.152	0.171	0.877	0.118	0.906
HDL-C	T_0_	1.14 ± 0.245	1.12 ± 0.21	T test	-0.072	0.111	0.021	0.429	0.669
	T_1_	1.20 ± 0.21	1.18 ± 0.23	T test	-0.059	0.119	0.200	0.666	0.507

LDL-C, Low-Density Lipoprotein Cholesterol; HDL-C, High-Density Lipoprotein Cholesterol. T_0_ is the baseline period. T_1_ is six months after the treatment.

In terms of weight management, the SGLT2i group showed a significant advantage (**Table 5**). At baseline, the two groups were comparable in all measured parameters. Following the 6-month treatment period, the SGLT2i group demonstrated a significantly greater reduction in body weight and body mass index than the DPP-4i group (*P* < 0.05). Regarding blood pressure management, SGLT2i therapy had a greater effect on systolic blood pressure (*P* < 0.05), whereas the improvement in diastolic pressure was comparable between the two groups.

**Table 5:** Comparison of changes in body weight and blood pressure indicators ( $\bar{x}$ ± s).

Parameters	SGLT2i group (n = 50)	DPP-4i group (n = 50)	Test	95%CI		Effect size	t	*P*-value
Weight (kg)	75.21 ± 5.32	77.54 ± 5.39	T test	-4.457	-0.203	0.176	-2.173	0.032
BMI (kg/m^2^)	27.54 ± 2.11	28.53 ± 1.58	T test	-1.720	-0.240	2.939	-2.628	0.010
Systolic blood pressure (mmHg)	133.31 ± 7.92	138.25 ± 8.02	T test	-8.101	-1.778	0.017	-3.101	0.003
Diastolic blood pressure(mmHg)	83.53 ± 4.79	84.82 ± 4.91	T test	-3.217	0.637	0.019	-1.328	0.187

BMI, Body Mass Index.

In terms of renal function protection, the SGLT2i also showed a significant advantage (**Table 6**). Following 6 months of therapy, the SGLT2i group achieved a significantly lower urinary albumin-to-creatinine ratio (UACR) than the DPP-4i group (*P* < 0.01), indicating better renal protection, despite the absence of a significant intergroup difference in the change of estimated glomerular filtration rate (eGFR) (*P* > 0.05).

**Table 6:** Comparison of renal function indicators ( $\bar{x}$ ± s).

Parameters		SGLT2i group (n = 50)	DPP-4i group (n = 50)	Test	95%CI		Effect size	t	*P*-value
UACR (mg/g)	T_0_	85.63 ± 15.34	84.21 ± 14.82	T test	-4.566	7.406	0.136	0.471	0.639
	T_1_	63.14 ± 12.81	78.93 ± 13.51	T test	-21.007	-10.553	0.014	-5.991	<.001
eGFR (ml/min/1.73m²)	T_0_	88.51 ± 10.17	87.78 ± 9.86	T test	-3.255	4.696	0.019	-0.360	0.720
	T_1_	86.33 ± 9.82	85.88 ± 9.56	T test	-3.396	4.296	0.428	0.232	0.817

UACR, Urine Albumin-to-Creatinine Ratio; eGFR, Estimated Glomerular Filtration Rate. T_0_ is Baseline period. T_1_ is Six months after the treatment.

The assessment of liver function safety (**Table 7**) showed that the liver function indicators of the two groups remained within the normal range during the treatment period. Among them, the SGLT2i group significantly outperformed the DPP-4i group in improving γ-glutamyl transferase (*P* < 0.05), with a decrease of (6.71 ± 4.2) U/L, which may be related to the effect of SGLT2i in improving metabolic fatty liver disease. All measured liver function parameters—specifically alanine aminotransferase, aspartate aminotransferase, alkaline phosphatase, total bilirubin, and albumin—showed no notable alterations between groups (*P* > 0.05) and consistently remained within the normal reference range. This outcome indicates that both medications were well tolerated from a hepatic perspective, with no evidence of hepatotoxicity, providing reassurance for their safe clinical use.

**Table 7:** Comparison of liver function indicators ( $\bar{x}$ ± s).

Parameters		SGLT2i group (n = 50)	DPP-4i group (n = 50)	Test	95%CI		Effect size	t	*P*-value
ALT(U/L)	T_0_	32.52 ± 8.31	31.85 ± 8.12	T test	-2.591	3.931	0.452	0.408	0.684
	T_1_	30.19 ± 7.06	30.53 ± 7.29	T test	-3.178	2.518	0.008	-0.230	0.819
AST (U/L)	T_0_	28.63 ± 6.49	27.95 ± 6.31	T test	-1.851	3.230	0.071	0.539	0.591
	T_1_	26.81 ± 5.94	27.12 ± 6.03	T test	-2.697	2.058	0.445	-0.267	0.790
ALP (U/L)	T_0_	85.62 ± 15.24	84.35 ± 14.46	T test	-4.696	7.236	0.136	0.422	0.674
	T_1_	82.33 ± 14.46	83.10 ± 14.62		-6.530	5.009	0.240	-0.261	0.794
γ-GGT	T_0_	45.21 ± 12.25	44.79 ± 11.94	T test	-4.382	5.223	0.739	0.174	0.863
	T_1_	38.50 ± 8.05	42.68 ± 9.25		-7.620	-0.741	0.011	-2.412	0.018
Total bilirubin (μmoL/L)	T_0_	12.83 ± 3.24	12.49 ± 3.12	T test	-0.924	1.604	0.016	0.534	0.595
	T_1_	13.09 ± 3.14	12.74 ± 3.09		-0.888	1.588	0.002	0.561	0.576
Albumin (g/L)	T_0_	42.48 ± 3.82	42.77 ± 3.56	T test	-1.755	1.175	0.544	-0.393	0.695
	T_1_	43.24 ± 3.62	42.96 ± 3.45		-1.135	1.676	0.063	0.382	0.703

ALT, Alanine aminotransferase; AST; Aspartate aminotransferase; ALP, Alkaline phosphatase; γ-GGT, γ-Glutamyl transferase. T_0_ is the baseline period. T_1_ is six months after the treatment.

After 6 months of treatment, the SGLT2i group significantly outperformed the DPP-4i group in the effect of high-sensitivity C-reactive protein (hs-CRP) (*P* < 0.05), suggesting its anti-inflammatory effect. At the same time, in terms of the improvement of central obesity indicators, such as waist circumference, the SGLT2i group also showed a significant advantage (*P* < 0.01), as shown in **Table 8**.

**Table 8:** Changes in inflammatory markers and other indicators ( $\bar{x}$ ± s).

Parameters		SGLT2i group (n = 50)	DPP-4i group (n = 50)	Test	95%CI		Effect size	t	*P*-value
hs-CRP (mg/L)	T_0_	3.84 ± 1.21	3.75 ± 1.09	T test	-0.357	0.557	0.731	0.433	0.666
	T_1_	2.55 ± 0.87	3.18 ± 0.96	T test	-0.992	-0.268	0.288	-3.458	0.001
Waist circumference (cm)	T_0_	96.53 ± 5.61	95.82 ± 5.75	T test	-1.543	2.963	0.170	0.626	0.533
	T_1_	93.16 ± 5.08	95.25 ± 5.18	T test	-4.126	-0.054	0.083	-2.037	0.044

hs-CRP, hypersensitive C-reactive protein. T_0_ is the baseline period. T_1_ is six months after the treatment.

### 3.4 Multivariate logistic regression analysis

This study used “glycemic treatment response” (a decrease of ≥1.0% in HbA1c after treatment) as the dependent variable and employed multivariate Logistic regression analysis to explore the independent predictors of treatment response. (**Table 9**). The results showed that after adjusting for potential confounding factors such as age, gender, duration of diabetes, baseline HbA1c, statin use, RAAS blockers, treatment group, diastolic blood pressure, history of hypertension, and total bilirubin, the following factors were independent predictors of glycemic treatment response (all *P* < 0.05): treatment group, diastolic blood pressure, history of hypertension, and total bilirubin.

**Table 9:** Multivariate Logistic regression analysis of independent risk factors.

Variable	B coefficient	Standard error	Wald χ²	*P*	OR	95% confidence interval
Treatment groups (SGLT2i vs DPP-4i)	0.558	0.219	6.489	0.011	1.747	1.137-2.684
Diastolic blood pressure (mmHg)	-0.190	0.089	4.557	0.033	0.827	0.694-0.985
History of hypertension (present vs absent)	-4.605	1.275	13.043	<0.001	0.010	0.001-0.121
Total bilirubin (μmol/L)	0.671	0.298	5.069	0.024	1.956	1.091-3.507
Age (years)	-0.015	0.012	1.563	0.211	0.985	0.962-1.009
Gender (Male vs Female)	0.189	0.165	1.312	0.252	1.208	0.874-1.670
Duration of diabetes (years)	-0.072	0.041	3.085	0.079	0.931	0.859-1.009
Baseline HbA1c (%)	0.142	0.108	1.728	0.189	1.153	0.933-1.425
Use of statin drugs (yes vs no)	0.213	0.176	1.464	0.226	1.237	0.876-1.747
Use of RAAS blockers (yes vs no)	0.187	0.169	1.224	0.269	1.206	0.866-1.679

The dependent variable is “glycemic treatment response”, defined as a decrease in glycated hemoglobin of ≥ 1.0% after treatment. Variables were included using the forward stepwise method. The Hosmer-Lemeshow goodness-of-fit test yielded a *p* value of 0.372, and the overall classification accuracy of the model was 78.0%.

Specifically, the likelihood of achieving a glycemic treatment response in patients in the SGLT2 inhibitor group was 1.747 times that in the DPP-4 inhibitor group (OR = 1.747, 95% CI: 1.137 - 2.684, pP = 0.011), suggesting that SGLT2 inhibitors are associated with a higher glycemic treatment response rate. Diastolic blood pressure was negatively correlated with treatment response, and for every 1 mmHg increase in diastolic blood pressure, the possibility of achieving treatment response decreased by 17.3% (OR = 0.827, 95% CI: 0.694 - 0.985, *P* = 0.033), indicating that patients with higher baseline diastolic blood pressure have greater treatment difficulty. History of hypertension was a strong negative predictor of treatment response, and patients with a history of hypertension had a 1.0% possibility of achieving treatment response compared to those without a history of hypertension (OR = 0.010, 95% CI: 0.001 - 0.121, *P* < 0.001), suggesting that hypertension is closely related to the difficulty in glycemic control. Total bilirubin was positively correlated with treatment response, and for every 1 μmol/L increase in total bilirubin, the possibility of achieving treatment response increased by 95.6% (OR = 1.956, 95% CI: 1.091 - 3.507, *P* = 0.024), suggesting that total bilirubin may be a protective biomarker involved in glycemic regulation.

The goodness-of-fit test of the model (Hosmer-Lemeshow test), *P* = 0.372, indicated that the model fits well, with a total classification accuracy rate of 78.0%, and could better predict the treatment response status of patients. The above results suggest that in clinical practice, choosing SGLT2 inhibitors for treatment, paying attention to the patient’s blood pressure status and history of hypertension, and assessing total bilirubin levels may help identify patient groups more likely to benefit from the treatment.

## 4 Discussion

Type 2 diabetes, as a complex chronic metabolic disorder, has seen a continuous increase in its global prevalence, and has become a major public health issue threatening human health (32, 33). Currently, diabetes management has shifted from merely controlling blood sugar levels to a comprehensive approach that also takes into account cardiovascular risk factors and target organ protection. This transformation has made the relative value assessment of new hypoglycemic drugs with different mechanisms of action a core issue of clinical concern (34). Among the various hypoglycemic drugs, SGLT2i and DPP-4 i have emerged as important options after the failure of metformin treatment due to their unique mechanism of action and favorable safety profile. However, the differences in clinical efficacy, safety, and benefits for specific patient groups between these two types of drugs remain a subject of debate in the academic community (35). This study, through a retrospective comparative analysis, aims to comprehensively evaluate the combined efficacy and safety profiles of these two types of drugs in real-world clinical settings, and to deeply explore the predictive factors influencing treatment responses, thereby providing evidence-based guidance for individualized clinical treatment.

In the treatment landscape of diabetes, SGLT2i and DPP-4i represent two fundamentally different therapeutic approaches. DPP-4i promotes insulin secretion in a glucose concentration-dependent manner by inhibiting the degradation of endogenous glucagon-like peptide-1. It is favored by clinicians due to its low risk of hypoglycemia, good safety and tolerability (36). SGLT2 inhibitors function by selectively blocking glucose reabsorption in the proximal renal tubules, thereby facilitating urinary glucose excretion and reducing blood glucose concentrations. This distinct, insulin-independent mode of action allows for consistent glycemic control in individuals with type 2 diabetes across various stages of disease progression (37). Recent large-scale cardiovascular outcome trials (CVOTs) have demonstrated that SGLT2 inhibitors possess cardioprotective properties extending beyond glucose-lowering efficacy. Notably, these agents show significant benefits in reducing the risk of heart failure hospitalization and slowing renal disease progression. These findings have substantially reshaped therapeutic strategies for type 2 diabetes (38). However, studies directly comparing their comprehensive benefits in real-world clinical settings are still relatively limited, and the conclusions are inconsistent, especially regarding the heterogeneity of treatment responses for specific patient groups, which has not been fully explored.

The innovation of this study lies in adopting an integrated research perspective and methodological strategy. Firstly, we constructed a multi-dimensional evaluation framework that combined primary efficacy indicators with multiple secondary outcome indicators, aiming to conduct a more comprehensive evaluation of the “comprehensive benefits” of the two types of drugs, rather than merely focusing on the single hypoglycemic effect. Secondly, compared to the simple “which drug is better” comparison, this study has taken a crucial step forward, aiming to explore the sources of treatment response heterogeneity by analyzing the association between baseline characteristics and treatment outcomes, and attempting to answer the more clinically practical question of “who is more likely to benefit more from which treatment”. Finally, this study focuses on recent real-world clinical data, which can reflect the actual application effects of these two types of drugs in the current clinical environment. The research findings of this study are expected to provide direct references for clinicians to formulate individualized treatment plans and promote the practical application of the precision medicine concept in the management of type 2 diabetes.

A detailed evaluation of the findings indicates that SGLT2i and DPP-4i demonstrate comparable effectiveness in glycemic management, with no statistically significant difference observed in HbA1c reduction between the two treatment groups. This outcome aligns with the clinical trial reported by Young Sang Lyu et al., further validating the efficacy of both drug classes as second-line antihyperglycemic agents (39). However, the similarity in the hypoglycemic effect does not imply the equivalence of overall efficacy. We observed significant differences in weight management, blood pressure control, and kidney protection. A more pronounced reduction in body weight was observed in the SGLT2i cohort compared to the DPP-4i group (*P* < 0.05). This finding aligns with the mechanisms of osmotic diuresis and caloric loss associated with SGLT2 inhibition, as described in the work of Patrick McLean et al. (40) What is more noteworthy is that the average systolic blood pressure in the SGLT2i group decreased by 4.35 mmHg, which was significantly better than 0.35 mmHg in the DPP-4i group. This difference carries considerable clinical relevance, as effective management of hypertension plays a vital role in mitigating cardiovascular risk among diabetic patients. Furthermore, regarding renal outcomes, the SGLT2i group exhibited a significantly greater improvement in the urinary albumin-to-creatinine ratio compared to the DPP-4i group. These renoprotective effects align with the established pharmacological mechanisms of SGLT2 inhibitors, which include ameliorating intraglomerular hypertension and attenuating inflammatory and fibrotic pathways (41, 42).

In terms of cardiovascular outcomes, this investigation observed a markedly reduced incidence of major adverse cardiovascular events in the SGLT2i cohort relative to the DPP-4i group. This result aligns with evidence from a 2023 comprehensive meta-analysis of cardiovascular outcome trials, thereby strengthening the premise of SGLT2 inhibitors’ cardioprotective efficacy (43). What is particularly noteworthy is that in recent years, studies by Miao Lin and others have also found that SGLT2i may have a protective effect on specific arrhythmias such as atrial fibrillation (44). A guideline jointly developed by the European Association for Cardio-Thoracic Surgery (EACTS) shows that the risk of atrial fibrillation in SGLT2i users is 43% lower than that in the DPP-4i group. This effect may be related to multiple mechanisms such as reducing atrial pressure load, improving myocardial energy metabolism, and regulating ion channel function (45). Furthermore, this study also found that SGLT2i was significantly superior to DPP-4i in terms of reducing triglycerides and improving high-sensitivity C-reactive protein, suggesting that SGLT2i may have broader metabolic regulation and anti-inflammatory effects. These effects may indirectly contribute to its cardiovascular protective benefits.

In terms of safety, the results of this study are largely consistent with the known safety characteristics of the two types of drugs. The incidence of genital infections in the SGLT2i group was higher, which is closely related to the pharmacological mechanism of this type of drug - the increase in glucose concentration in urine provides a favorable environment for microbial growth (46, 47). However, through appropriate patient education and preventive measures, most genital infections can be effectively managed and prevented. Notably, the incidence of hypoglycemia was similarly low in both treatment groups, indicating that both drug classes exhibit a glucose-dependent mechanism of action with a favorable safety profile regarding hypoglycemic risk. Additionally, long-term cancer risk is also an important consideration in evaluating the safety of hypoglycemic drugs. A meta-analysis of cohort studies showed that compared with DPP-4i, the overall cancer risk of SGLT2i was significantly reduced (combined risk ratio = 0.77, 95% confidence interval: 0.70 - 0.84). Subgroup analysis revealed that SGLT2i showed significant effects in reducing the risks of liver cancer (risk ratio = 0.76), lung cancer (risk ratio = 0.87), and prostate cancer (risk ratio = 0.75) (48). These findings provide support for the long-term safety of SGLT2i, but further validation is needed through randomized controlled trials with longer follow-up periods.

This study used multivariate Logistic regression analysis to explore the independent predictors of treatment response in patients with type 2 diabetes. The criterion for “glycemic treatment response” was defined as a decrease of ≥1.0% in HbA1c after treatment. This outcome variable was selected based on its clear clinical significance and research value. From a clinical perspective, a 1% reduction in HbA1c has been confirmed by multiple large-scale studies to be closely related to a 30% reduction in the risk of microvascular complications. This standard not only has statistical significance but is also representative of substantial clinical benefits (49). From the perspective of research methodology, converting continuous variables into binary classification variables helps to enhance the clinical interpretability of the results, enabling doctors to make more intuitive judgments on the success of treatment. This also aligns with the thinking mode of clinical decision-making in the real world.

Analysis revealed that treatment with SGLT2 inhibitors was identified as a significant independent predictor of glycemic response. The adjusted odds ratio for achieving target glycemic control was 1.747 compared to DPP-4 inhibitors, a finding closely associated with the distinct pharmacological profile of SGLT2 inhibitors. Unlike the hypoglycemic mechanism of DPP-4i that relies on the function of pancreatic β cells, SGLT2i exerts its hypoglycemic effect by urinary glucose excretion. This mechanism does not depend on insulin secretion, so it maintains relatively stable efficacy in various types of type 2 diabetes patients. This result is consistent with the significant hypoglycemic effect of SGLT2i mentioned in the article by Zhi-Cheng Dai et al., further verifying its superior position in glycemic control (50).

What is particularly noteworthy is that both diastolic blood pressure and the history of hypertension show a negative correlation with the response to blood sugar treatment. For every one-unit increase in diastolic blood pressure, the likelihood of achieving a blood sugar treatment response decreases by 17.3%. Patients with a history of hypertension exhibited a treatment response rate that was merely 1% of that observed in non-hypertensive patients. This finding reveals the intrinsic connection between hypertension and the difficulty in controlling blood sugar. From a pathophysiological perspective, this may be related to the fact that patients with hypertension often have a more severe insulin resistance state. Characteristics of hypertension such as excessive activation of the sympathetic nervous system and abnormal function of the renin-angiotensin system may interfere with insulin signal transduction, thereby affecting the therapeutic effect of hypoglycemic drugs (51). This finding is in line with the observation in the ACCORD study that blood sugar control and blood pressure management have an interactive effect. It suggests that for diabetic patients with hypertension, when choosing a hypoglycemic strategy, a more comprehensive management approach should be emphasized (52).

Total bilirubin, as a biomarker with antioxidant properties, has a particularly significant positive correlation with the response to glucose treatment. For every one-unit increase in total bilirubin, the likelihood of achieving a treatment response increases by 95.6%. This finding provides a new perspective for understanding glucose regulation. Recent studies have shown that bilirubin is not only a metabolic product but also has significant antioxidant and anti-inflammatory properties, which can improve insulin resistance through mechanisms such as eliminating free radicals and inhibiting oxidative stress (53). Our findings align with multiple cross-sectional investigations that report an inverse relationship between serum bilirubin concentrations and diabetes prevalence. These collective observations lend further support to the proposed beneficial role of bilirubin in glucose homeostasis (54). This discovery suggests that total bilirubin may serve as a valuable biomarker to help identify patient groups that respond better to hypoglycemic treatment.

From a deeper mechanistic perspective, the influence of SGLT2i and DPP-4i on treatment response heterogeneity may stem from their different regulatory effects on various physiological pathways. In addition to the known hypoglycemic and diuretic effects, SGLT2i may also exert organ-protective effects through multiple mechanisms such as promoting ketone body production to improve myocardial energy supply, inhibiting myocardial sodium-hydrogen exchangers, and reducing inflammation and fibrosis in renal and vascular tissues (55). In addition to inhibiting the DPP-4 enzyme and increasing the level of endogenous GLP-1, DPP-4i may also affect the metabolism of other various bioactive peptides, such as stromal cell-derived factor-1. This may play a role in tissue repair and inflammation regulation (56). These complex mechanism networks may have different net effects due to the patient’s basal metabolic state, genetic background and complication conditions, thereby explaining the heterogeneity of treatment responses.

It is worth noting that the patient’s treatment preferences can also affect the actual efficacy and compliance of the drugs. A study on the treatment preferences of Asian patients with type 2 diabetes found that 64.5% of the patients significantly preferred SGLT2i with cardiovascular and renal protective effects, with the main considerations including better blood sugar control, reduced risk of heart failure hospitalization, and improved risk of kidney disease (57). This preference is closely related to the clinical characteristics of the patients. Women, overweight patients, and those with hypertension, dyslipidemia, and chronic kidney disease are more likely to choose SGLT2i. Understanding the patients’ preferences and their influencing factors is helpful for clinicians to communicate better with the patients during the shared decision-making process, thereby improving long-term treatment compliance.

For diabetic patients with mental health problems, drug selection also requires special caution. A study by Riccardo De Giorgi et al. compared the neuro-psychiatric outcomes of semaglutide (a GLP-1 receptor agonist) with other hypoglycemic drugs (including DPP-4i and SGLT2i) in patients with type 2 diabetes. The study found that semaglutide did not increase the risk of neuro-psychiatric events, and showed potential benefits in certain aspects (such as cognitive impairment and nicotine abuse), providing a guarantee for the medication safety of this special population (58).

Although studies have confirmed the individual hypoglycemic effects and safety profiles of SGLT2 inhibitors and DPP-4 inhibitors, there are still relatively limited direct comparative studies in real-world clinical settings that assess the combined benefits of both. Most of these studies focus on a single efficacy indicator and lack a systematic evaluation of multiple outcomes (including weight, blood pressure, renal function, cardiovascular events, etc.). More importantly, there is currently insufficient evidence and consensus regarding which baseline characteristics can predict the treatment response of these two types of drugs in different patients. This research gap limits the precise selection of clinicians in individualized treatment decisions. Therefore, this study aims to conduct a retrospective cohort analysis to systematically compare the clinical efficacy and safety of SGLT2 inhibitors and DPP-4 inhibitors in treating patients with type 2 diabetes, and further explore the independent predictors of treatment response, in order to provide evidence-based guidance for individualized clinical treatment.

In reviewing the clinical significance of this study, it further reinforces the role of SGLT2i in the treatment of type 2 diabetes, especially for patients with overweight/obesity, hypertension, high cardiovascular disease risk, or early diabetic nephropathy. At the same time, the research results also support the continued use of DPP-4i in specific patient groups, particularly those with a shorter disease course, higher post-meal blood glucose levels, or those who cannot tolerate the adverse reactions associated with SGLT2i. Most importantly, this study emphasizes the importance of individualized treatment decisions. Clinicians should select the most suitable hypoglycemic drugs based on the patient’s baseline characteristics, complications, treatment preferences, and drug safety profiles, rather than adopting a “one-size-fits-all” treatment strategy.

## 5 Limitations

This study has several limitations. Firstly, this study is a single-center retrospective design, and all data are derived from the same hospital. The patient population may have regional characteristics and preferences regarding diagnostic and treatment methods, which limit the generalizability of the research results. There may be differences in the patient’s composition, diagnostic protocols, and follow-up systems among different medical institutions. Therefore, the applicability of this conclusion in other centers or a broader population still needs to be further verified. In the future, multicenter prospective studies are needed to validate this study’s findings and enhance the external validity of the results.

Secondly, as a retrospective study, although we attempted to control known confounding factors through multivariate regression analysis (including demographic characteristics, clinical features, comorbidities, and concomitant medications, etc.), we still could not completely rule out the potential influence of unmeasured confounding factors (such as lifestyle, dietary control, treatment compliance, etc.) on the results. Moreover, the sample size of this study is relatively small, and the follow-up period is short, resulting in insufficient power to assess rare adverse events. Long-term efficacy and safety need to be further observed.

Furthermore, this study did not distinguish between different specific subtypes of SGLT2i and DPP-4i. However, different drugs may differ in their molecular structures, pharmacological properties, and clinical effects. Additionally, some patients received combination therapy, and the efficacy of different regimen may vary. Due to the limited sample size, this study was unable to conduct a subgroup analysis of different combination regimens. Larger-sample-size studies are needed in the future to further verify these findings.

It should be noted that the long-term use of certain medications (such as statins, RAAS blockers, etc.) may have an independent impact on cardiovascular outcomes. In our analysis, we have included this as a covariate in the model to control for its confounding effect on the primary outcome.

Future research should further validate these findings through prospective designs with larger sample sizes and longer follow-up periods, and conduct an in-depth exploration of the predictive value of genetic factors, biomarkers, and new imaging indicators for treatment responses. At the same time, conducting intervention studies based on predictive models to evaluate the effectiveness and cost-effectiveness of individualized treatment strategies in actual clinical practice will also be an important research direction.

## 6 Conclusion

In conclusion, this study conducted a comprehensive comparison of the efficacy and safety of SGLT2i and DPP-4i in real-world clinical settings, and identified key predictors of treatment response. These findings provide valuable evidence-based support for individualized treatment of type 2 diabetes. The results suggest that in the treatment decisions for patients with type 2 diabetes, consideration should be given to the patient’s baseline characteristics, complications, and personal preferences, in order to select the most suitable hypoglycemic drugs, aiming to achieve the dual goals of blood glucose control and organ protection, and ultimately improving the long-term prognosis of patients. With the deepening practice of precision medicine in the field of diabetes, in the future, we expect to see more individualized treatment strategies based on predictive models, enabling each patient to receive the treatment plan most suitable for their own characteristics, thereby maximizing the treatment benefit-to-risk ratio.

## Data Availability

The original contributions presented in the study are included in the article/supplementary material. Further inquiries can be directed to the corresponding author.
